# LncRNA SNHG16 promotes pulmonary fibrosis by targeting miR-455-3p to regulate the Notch2 pathway

**DOI:** 10.1186/s12931-021-01632-z

**Published:** 2021-02-06

**Authors:** Panpan Liu, Lei Zhao, Yuxia Gu, Meilan Zhang, Hongchang Gao, Yingxia Meng

**Affiliations:** Department of Pulmonary and Critical Care Medicine, Shanghai Pudong New Area Gongli Hospital, 219 Miao Pu Road, Shanghai, 200315 China

**Keywords:** LncRNA SNHG16, Pulmonary fibrosis, MiR-455-3p, Notch2

## Abstract

**Background:**

Idiopathic pulmonary fibrosis (IPF) is the most common interstitial lung diseases with a poor prognosis. Long non-coding RNAs (lncRNAs) have been reported to be involved in IPF in several studies. However, the role of lncRNA SNHG16 in IPF is largely unknown.

**Methods:**

Firstly, experimental pulmonary fibrosis model was established by using bleomycin (BML). Histology and Western blotting assays were used to determine the different stages of fibrosis and expression of several fibrosis biomarkers. The expression of SNHG16 was detected by quantitative real-time polymerase chain reaction (qRT‐PCR). EdU staining and wound-healing assay were utilized to analyze proliferation and migration of lung fibroblast cells. Molecular mechanism of SNHG16 was explored by bioinformatics, dual-luciferase reporter assay, RNA immunoprecipitation assay (RIP), and qRT-PCR.

**Results:**

The expression of SNHG16 was significantly up-regulated in bleomycin-(BLM) induced lung fibrosis and transforming growth factor-β (TGF-β)-induced fibroblast. Knockdown of SNHG16 could attenuate fibrogenesis. Mechanistically, SNHG16 was able to bind and regulate the expression of miR-455-3p. Moreover, SNHG16 also regulated the expression of Notch2 by targeting miR-455-3p. Finally, SNHG16 could promote fibrogenesis by regulating the expression of Notch2.

**Conclusion:**

Taken together, our study demonstrated that SNHG16 promoted pulmonary fibrosis by targeting miR-455-3p to regulate the Notch2 pathway. These findings might provide a novel insight into pathologic process of lung fibrosis and may provide prevention strategies in the future.

## Background

Over the past decade, the incidence of idiopathic pulmonary fibrosis (IPF) is gradually increasing globally [1]. IPF generally affects men after the age of 60 years, and the prognosis of patients with IPF is extremely poor, with a median survival time of 2–4 years, even worse than many cancers [2, 3]. Pathologically, IPF is characterized as a chronic, progressive fibrotic parenchymal lung disorder with excessive deposition of extracellular matrix (ECM) [4]. Accumulating evidences suggest that plenty of mechanisms contribute to pathological change of IPF, such as epithelial injury and activation, inflammation, and abnormal remodeling [4–7]. Despite great achievements have been made in study of pathological changes in IPF, little is known about the origin of activation of fibroblasts during fibrotic remodeling. A better understanding of the mechanism is greatly needed.

Non-protein coding RNAs (ncRNAs) account for the majority of RNA, and only approximately 1.9% of RNAs encode proteins [8, 9]. Previously, ncRNAs were recognized as “evolutionary junk,” without any biological functions. However, with the development of the deep sequencing technology, accumulating evidences indicate that ncRNAs have great impacts on molecular mechanisms of animals and even human [10–12]. Generally, ncRNAs can be divided into two categories based on size, and longer than 200 nucleotides (nt) are classified as long non-coding RNAs (lncRNAs), whereas smaller than 200 nucleotides are known as short non-coding RNAs, e.g. microRNAs (miRNAs), repetitive RNAs and intronic RNAs [13, 14]. Among short non-coding RNAs, miRNAs, with a size of ∼20 nt, have attracted widespread attention [15]. Reports have demonstrated that several lncRNAs and miRNAs play critical roles in the progression of IPF. For instance, Savary et al. reported that lncRNA DNM3OS played an important role in TGF-β-induced lung myofibroblast activation by regulating several miRNAs [16]. Another lncRNA pulmonary fibrosis-associated (PFAL) was highly expressed in lung fibrosis tissue of experimental mice. In TGF-β1-induced fibrotic lung fibroblasts, PFAL could promote cell proliferation, migration, and fibroblast-myofibroblast transition by regulating miR-18a [17]. In addition, lncRNA H19 [18], MEG3 [19] have been also confirmed to paly critical roles in IPF.

Previously, a large number of studies have focused on a certain lncRNA called SNHG16, which was reported to promote epithelial-mesenchymal transition (EMT) of many type of cancer cells [20, 21]. More importantly, it could also promote proliferation and migration of lung cancer cells [22]. However, whether SNHG16 plays regulatory roles in lung fibrosis remains unknown and still needs to be explored.

In the present study, we used both BLM-induced pulmonary fibrotic animal model and TGF-β1-induced pulmonary fibrosis in vitro model to explore the role of lncRNA SNHG16 in the progression of pulmonary fibrosis. The aim of this study was to clarify the regulatory role of SNHG16 and mechanisms of its down-stream factors in pulmonary fibrosis. Our study not only sets a novel sight into pathophysiological mechanism of IPF, but also provides a theoretical basis for the research of new therapeutic target for IPF.

## Methods

### Experimental pulmonary fibrosis model

All the animal-related experiments were performed based on the Ethics Committees of Shanghai Pudong New Area Gongli Hospital. C57BL/6 mice (male, 6–8 weeks old) were obtained from Charles River Animal Technology (Beijing, China). To establish the pulmonary fibrosis model, bleomycin (BLM, Sigma-Aldrich, Billerica, MA, USA) dissolved in saline was intratracheally administered (1.5 U/kg of body weight) under anesthesia. Equal volume of sterile saline was injected in mice which were used for control. In another series of experiments, 72 h after administration of BLM, mice were intratracheally injected with adenovirus-associated short hairpin RNA (shRNA) (VectorBuilder, Guangzhou, China) for lncRNA SNHG16 (Ad-sh-SNHG16#1, Ad-sh-SNHG16#2) or its control (Ad-sh-NC). Mice were sacrificed on the 28th day after BLM administration.

### Histologic experiments

The pulmonary tissues were fixed in 4% paraformaldehyde solution for 7 days. After dehydration, tissues were embedded in paraffin for histopathological analysis. Sections were prepared with a thickness of 4 μm and stained with hematoxylin and eosin (H&E) or Masson’s trichrome kit (Shanghai, China) based on manufacturers’ instructions.

### Immunohistochemistry (IHC)

Briefly, paraffin sections were treated with 0.3% H_2_O_2_ in methanol. Afterwards, sections were incubated with primary antibody Anti-α-SMA (1:1000, #ab32575, Abcam, Cambridge, MA, UK) overnight at 4 °C. The following day, goat anti-rabbit HRP labeled secondary antibody was added, and then immunostained using DAB plus kit. Images were acquired under a fluorescence microscope (Leica, Germany).

### Western blotting

Proteins from different groups of lung tissues or fibroblasts were extracted using RIPA lysis buffer (Sigma, USA) and qualified by a BCA kit (Beyotime Biotechnology, Nanjing, China). Then, samples were separated by electrophoresis, transferred onto a PVDF membrane (Invitrogen, USA). After incubated in 5% blocking buffer, membranes were incubated with primary antibodies at 4 °C overnight: α-SMA (1:500, #ab7817, Abcam), E-cadherin (1:1000, # ab40772, Abcam), collagen 1 (1:1000, # ab34710, Abcam), fibronectin 1 (1:1000, # ab2413, Abcam), Notch2 (1:1000, # ab8926, Abcam), GADPH (1:1000, # ab8245, Abcam). The next day, secondary antibodies were added and incubated for 40 min. Images were detected using the Odyssey Infrared Imaging System.

### Quantitative real-time PCR (qRT-PCR)

Total RNA was extracted from lung tissues or cultural lung fibroblasts using TRIzol reagent (Invitrogen, USA). cDNA was synthesized by reverse transcriptase kit (Takara, Otsu, Japan). Gene expression was determined with SYBR Green I on a ABI 7500 fast Real-Time PCR instrument (California, USA). The primer sequences for SNHG16, GAPDH, miR-455-3p, U6 were listed in Table 1. Relative mRNA levels and miRNA were calculated based on the Ct values and normalized to the GAPDH or U6 expression in each sample, respectively. Data were analyzed using the 2^−ΔΔCT^ method.

**Table 1:** Primers used in the present study

Gene	Primer sequence
miR-455-3p	forward: 5′-ATAAAGRGCRGACAGTGCAGATAGTG-3′
	reverse: 5′-TCAAGTACCCACAGTGCGGT-3′
U6	forward: 5′-GCTTCGGCAGCACATATACT-3′
	reverse: 5′-GTGCAGGGTCCGAGGTATTC-3′
SNHG16	forward: 5′-CCAAGCTTATGCCAGATGGGATCAGCAC-3′
	reverse: 5′-CCGCTCGAGCTTGGTGAGTCAACACTGGGT-3′
GAPDH	forward: 5′-GGAGCGAGATCCCTCCAAAAT-3′ reverse: 5′-GGCTGTTGTCATACTTCTCATGG-3′

### Cell culture and treatment

Primary mouse lung fibroblasts were isolated from 2-day-old C57BL/6 mice. The lung tissue was removed and digested with 0.25% trypsin. Isolated cells were washed and resuspended in DMEM (Gibco, Grant Island, NY, USA) supplemented 10% fetal bovine serum (Gibco, USA), and 1% penicillin G/streptomycin (Beyotime Institute of Biotechnology, Nanjing, China). Lung fibroblasts were cultured at 37 °C with 5% CO_2_. For treatment, recombinant human TGF-β1 (10 ng/ml; PeproTech, USA) was used for establishing pulmonary fibrosis model in vitro.

### Cell transfection

Prior to TGF-β1 treatment, lung fibroblasts were treated with serum-free culture medium for 5 h. Then, lentivirus-mediated short hairpin RNA (shRNA) target SNHG16 or its negative control (sh-NC), miR-455-3p mimics or inhibitor and their control plasmids (NC mimic, NC inh), pcDNA3.1( +) Notch2 Vector for overexpressing Notch2 and its control (mock) purchased from GenePharma (Shanghai, China) were transfected into lung fibroblasts using lipotectamine 2000 (Invitrogen, Carlsbad, CA, USA) based on manufacturer’s instructions.

### Immunofluorescence staining

Transfected lung fibroblasts were fixed with 4% paraformaldehyde solution. After permeabilization and blocking treatment, cells were incubated with primary antibody against mouse α-ASM (1:500, # ab83354, Abcam) overnight at 4 °C. Next, sections were incubated with fluorescein isothiocyanate (FITC)-conjugated goat anti-mouse in dark. Finally, cells were stained with diamidino-2-phenylindole (DAPI; 1:1000; Beyotime, Nanjing, China) for 15 min. Data were acquired and analyzed using a fluorescence microscope (Nikon 80i, Tokyo, Japan).

### Cell proliferation assays

EdU staining was used for detecting cellular proliferation. Briefly, lung fibroblasts were cultured in 24-well plates. Cell-Light EdU DNA Cell Proliferation Kit (RiboBio, Guangzhou, China) were used for detecting cell proliferation based on manufacturer’s instructions.

### Wound-healing assay

Different groups of transfected lung fibroblast cells were seeded into 6-well culture plates and grew until ~ 80% confluence. The cell monolayer was wounded by scratching with a 200 µl pipette tip. The movement of cells was calculated using an olympus microscope at 0 and 24 h.

### Dual-luciferase reporter assay

Putative wild-type (WT) and mutant (Mut) miR-455-3p binding sites in the 3ʹ-UTR of SNHG16 (SNHG16-WT, SNHG16-Mut) and Notch2 (Notch2-WT, Notch2-Mut) were cloned into a pmirGLO-Report luciferase vector (Promega, Madison, WI, USA). HEK293 cells were transfected with SNHG16-WT or SNHG16-Mut, as well as Notch2 3′UTR WT or Notch2 3′UTR Mut, followed transfecting with miR-455-3p mimics or NC mimics using Lipofectamine 3000 (Invitrogen, USA). An amount of 5 ng/well Renilla luciferase plasmid was used as internal control. Luciferase activity was measured with the Dual-Luciferase Reporter Assay System (Promega, USA) 48 h after transfection.

### RNA binding protein immunoprecipitation (RIP) assay

The RIP assay was used to explore the binding relationship between endogenous SNHG16 and miR-455-3p. Briefly, lysate of transfected lung fibroblasts was treated with RIP buffer containing magnetic beads conjugated with anti-Ago2 antibody (Millipore, Billerica, MA, USA), or negative control IgG. Wash buffer was used to wash the beads, and the complexes were incubated with 0.1% SDS/proteinase K to remove proteins. qRT-PCR assay was performed thereafter.

### Statistical analysis

All experiments were repeated in triplicate. Data were exhibited as mean ± SD. A one-way ANOVA followed by Dunnett’s test was used for multiple comparisons. A two-tailed *p* value less than 0.05 was considered as statistically significant difference. Statistical analyses were carried out using the GraphPad Prism 7.0 and Statistical Package for Social Sciences version 21.0 (SPSS, Inc., Chicago, IL).

## Results

### SNHG16 was highly expressed in BLM-induced pulmonary fibrotic tissues

**Fig. 1:**
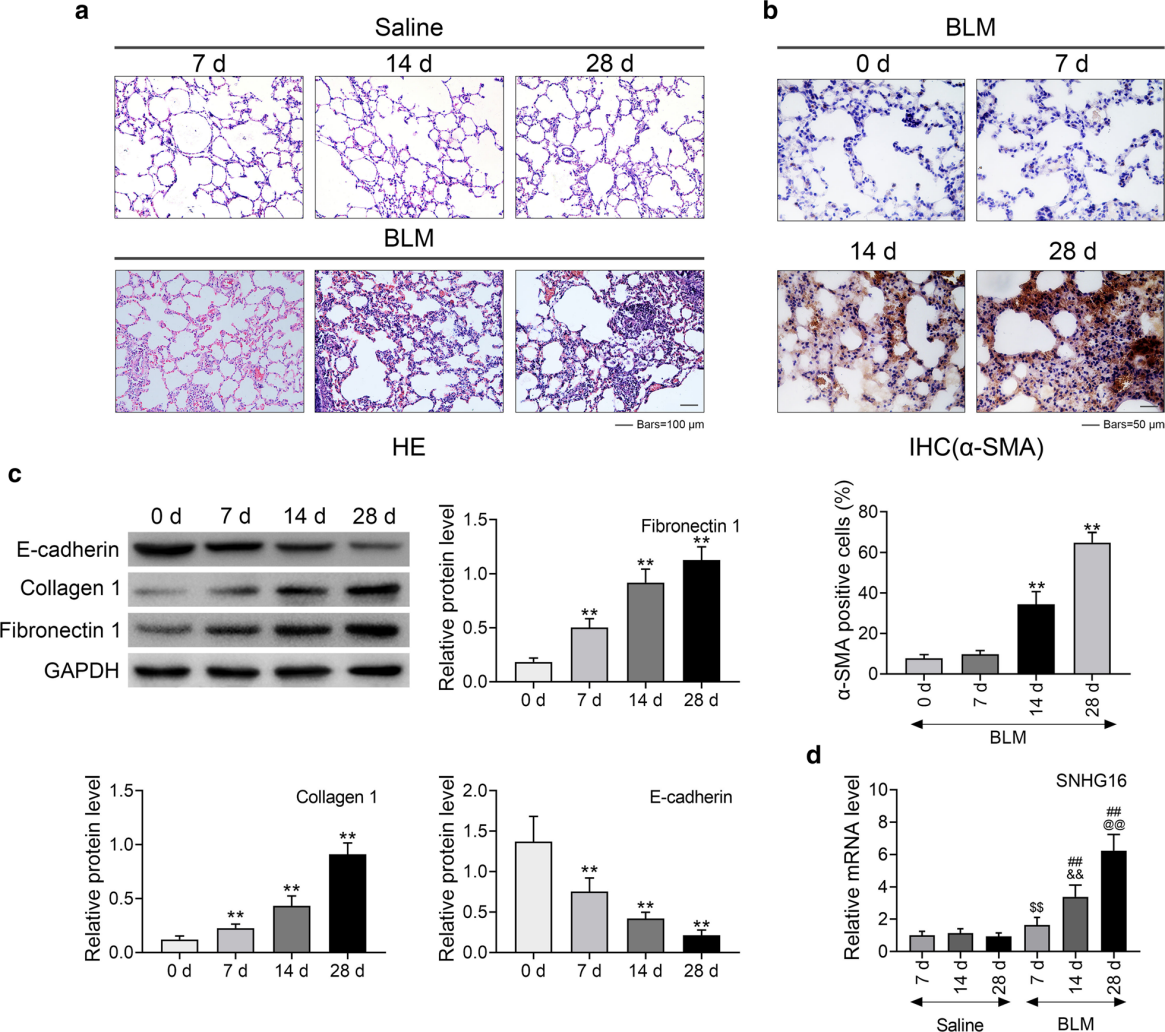
SNHG16 was highly expressed in BLM-induced pulmonary fibrotic tissues. **a** BLM-induced pulmonary fibrotic tissues were checked by HE-staining (7 days, 14 days and 28 days after BLM injection). **b** The expression of α-SMA was detected by IHC (0 day, 7 days, 14 days and 28 days after BLM injection). **c** The expression level of E-cadherin, collagen 1 and fibronectin 1 in pulmonary fibrotic tissues were determined by western blot (7 days, 14 days and 28 days after BLM injection). **d** The expression level of SNHG16 was determined by qRT-PCR (7 days, 14 days and 28 days after BLM injection). Data were expressed as mean ± SD. Six mice were used in each group (n = 6) **P < 0.01 vs baseline, ^$$^*P* < 0.01 vs 7 days saline; ^##^*P* < 0.01 vs 7 days BLM; ^&&^*P* < 0.01 vs 14 days saline; ^@@^*P* < 0.01 vs 28 days saline, represent statistically difference

First of all, the pulmonary fibrotic model was established by BLM injection. Lung tissues were examined by HE-staining at 7 days, 14 days and 28 days after BLM injection. As shown in Fig. 1a, in BLM-induced pulmonary fibrotic tissues, alveolar and blood barrier structures were damaged in a time-dependent manner. Inflammatory cell infiltration, diffused pulmonary fibrosis and fibrotic nodule formation were observed in BLM group. In addition, expression level of α-SMA was also shown in a time-dependent manner, which was detected by IHC (Fig. 1b). Moreover, expression of E-cadherin, collagen 1 and fibronectin 1 were determined by western blot at 7 days, 14 days and 28 days after BLM injection. Results demonstrated that the expression of E-cadherin was significantly decreased overtime (Fig. 1c, p < 0.01), while the expression of collagen 1 and fibronectin 1 was dramatically increased overtime (Fig. 1c, P < 0.01). All the results above indicated that pulmonary fibrosis model was successfully created. Finally, the relative expression of SNHG16 mRNA in lung tissues was determined by qRT-PCR in saline or BLM group, respectively. And the results showed that the expression of SNHG16 mRNA was up-regulated at 7 days, 14 days and 28 days after BLM injection compared with the corresponding time points of control (Fig. 1d, p < 0.01). Moreover, the expression of SNHG16 mRNA in BLM group was significantly increased in overtime (Fig. 1d, p < 0.01).

### Knockdown of SNHG16 attenuated BLM-induced pulmonary fibrosis

**Fig. 2:**
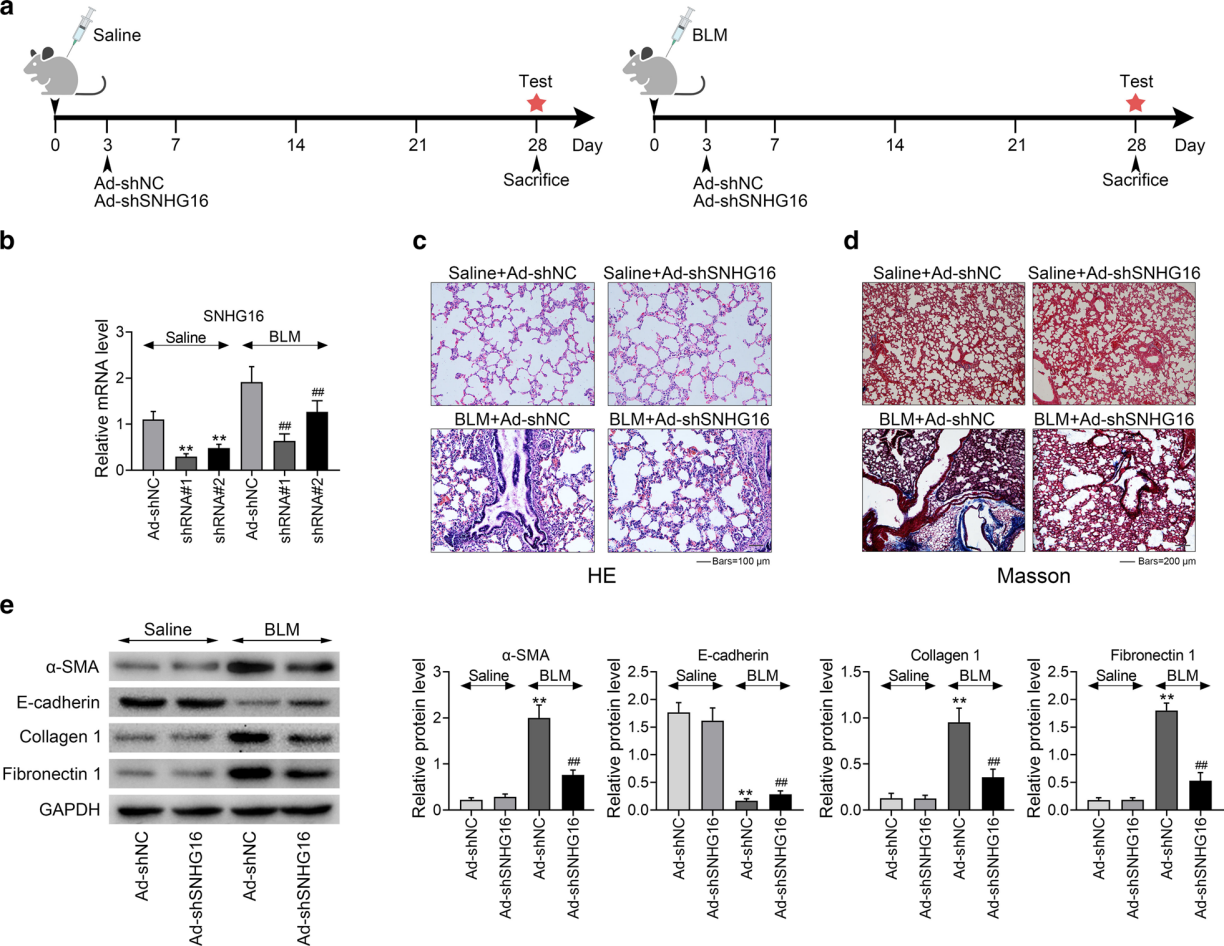
Knockdown of SNHG16 attenuated BLM-induced pulmonary fibrosis. **a** The timelines of both groups were shown. **b** The expression level of SNHG16 were measured by qRT-PCR in saline or BLM injected mice which treated with shRNAs targeted SNHG16 (shSNHG16#1, shSNHG16#2). **c**, **d** HE and Massion staining were performed on lung tissues (saline or BLM injected) with sh-SNHG16 treated or its control (sh-NC). **e** The expression level of protein α-SMA, E-cadherin, collagen 1 and fibronectin 1 in BLM injected or saline injected group with or without knockdown of SNHG16 were determined by western blot. Data were expressed as mean ± SD. **P < 0.01 vs Ad sh-NC Saline, ^##^*P* < 0.01 vs Ad sh-NC BLM, represent statistically difference

To investigate the role of SNHG16 in pulmonary fibrosis, we used shRNA which specifically targets SNHG16 to knockdown the expression of SNHG16. Timelines of both groups were shown in Fig. 2a. Then, the expression of SNHG16 was significantly down-regulated in both saline and BLM injected group (Fig. 2b, p < 0.01). Moreover, shRNA#1 had more profound effect which was selected to be used in further experiments. Next, histology tests include HE and Massion staining were performed on lung tissues of different groups. As shown in Fig. 2c, d, 28 days after BLM injection, knockdown of SNHG16 could significantly suppress the fibrogenesis of lung tissues. No change was detected in saline injected group. Furthermore, the expression of α-SMA, E-cadherin, collagen 1 and fibronectin 1 in BLM injected or saline injected group with or without knockdown of SNHG16 were determined by western blot. Results demonstrated that the expression of α-SMA, collagen 1 and fibronectin 1 were significantly increased in BLM injected group, while the level of these proteins were suppressed by silencing SNHG16 (Fig. 2e, p < 0.01). On the contrary, the expression of E-cadherin was remarkably decreased in BLM injected group compared with control group. Moreover, its level can be up-regulated by silencing SNHG16 (Fig. 2e, p < 0.01).

### Knockdown of SNHG16 inhibited fibrogenesis of lung fibroblast cells

**Fig. 3:**
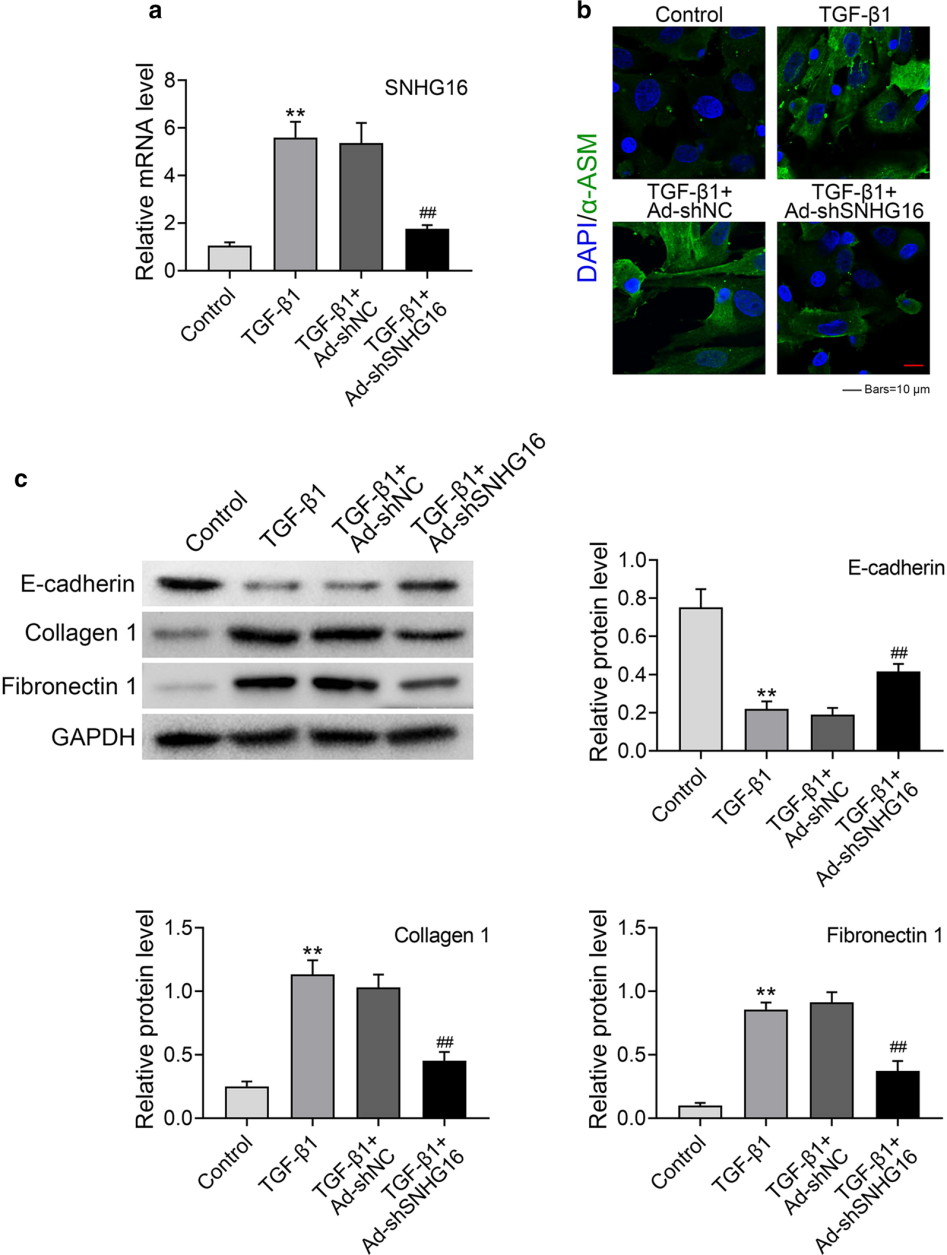
Knockdown of SNHG16 inhibited fibrogenesis of lung fibroblast cells. TGF-β1 treated lung fibroblast cells which transfected with sh-SNHG16 or sh-NC were used for experiments: **a** relative mRNA levels of SNHG16 in different groups were determined by qRT-PCR. **b** The expression levels of α-SMA in different groups were checked by IF. **c** The expression of E-cadherin, collagen 1 and fibronectin 1 in different groups were measured by western blot. Data were expressed as mean ± SD. **P < 0.01 was control, ^##^*P* < 0.01 vs TGF-β1 + Ad sh-SNHG16, represent statistically difference

To further explore the underlying mechanism of SNHG16 in lung fibrogenesis, we used primary mice lung fibroblast cells (isolated from C57BL/6 mice) to conduct a series of experiments. TGF-β1 (5 ng/ml) was used to induce the lung fibroblast. sh-SNHG16 or its control sh-NC was transfected into mouse lung fibroblast to downregulate the expression of SNHG16. Then, the expression o level of SNHG16 in mouse lung fibroblasts was tested. As shown in Fig. 3a, the expression of SNHG16 mRNA was significantly increased in TGF-β1 induced group, while knockdown of SNHG16 could inhibit the expression of SNHG16 (Fig. 3a, p < 0.01), suggesting that this system can be used in further studies. Then, α-ASM, a bio-marker of lung fibrogenesis was detected by immunofluorescence staining in lung fibroblast cells. The results demonstrated that the expression of α-ASM significantly increased in lung fibroblast cells treatment of TGF-β1, however, this effect can be reversed by knocking down of SNHG16, indicating that silence of SNHG16 could inhibit fibrogenesis of lung fibroblast cells (Fig. 3b). Furthermore, the expression levels of E-cadherin, Collagen 1 and Fibronectin 1 in different groups were determined by western blot. As shown in Fig. 3c, the expression of E-cadherin was dramatically down-regulated after the cells treated with TGF-β1, whereas silence of SNHG16 up-regulated the expression of E-cadherin (Fig. 3c, p < 0.01). On the contrary, the expression levels of Collagen 1 and Fibronectin 1 were remarkably increased in TGF-β1 treated group, while knockdown of SNHG16 down-regulated the expression of these two factors (Fig. 3c, p < 0.01). Taken together, these results suggested that knockdown of SNHG16 could inhibit fibrogenesis of lung fibroblast cells.

### Knockdown of SNHG16 suppressed the proliferation and migration of lung fibroblast cells

**Fig. 4:**
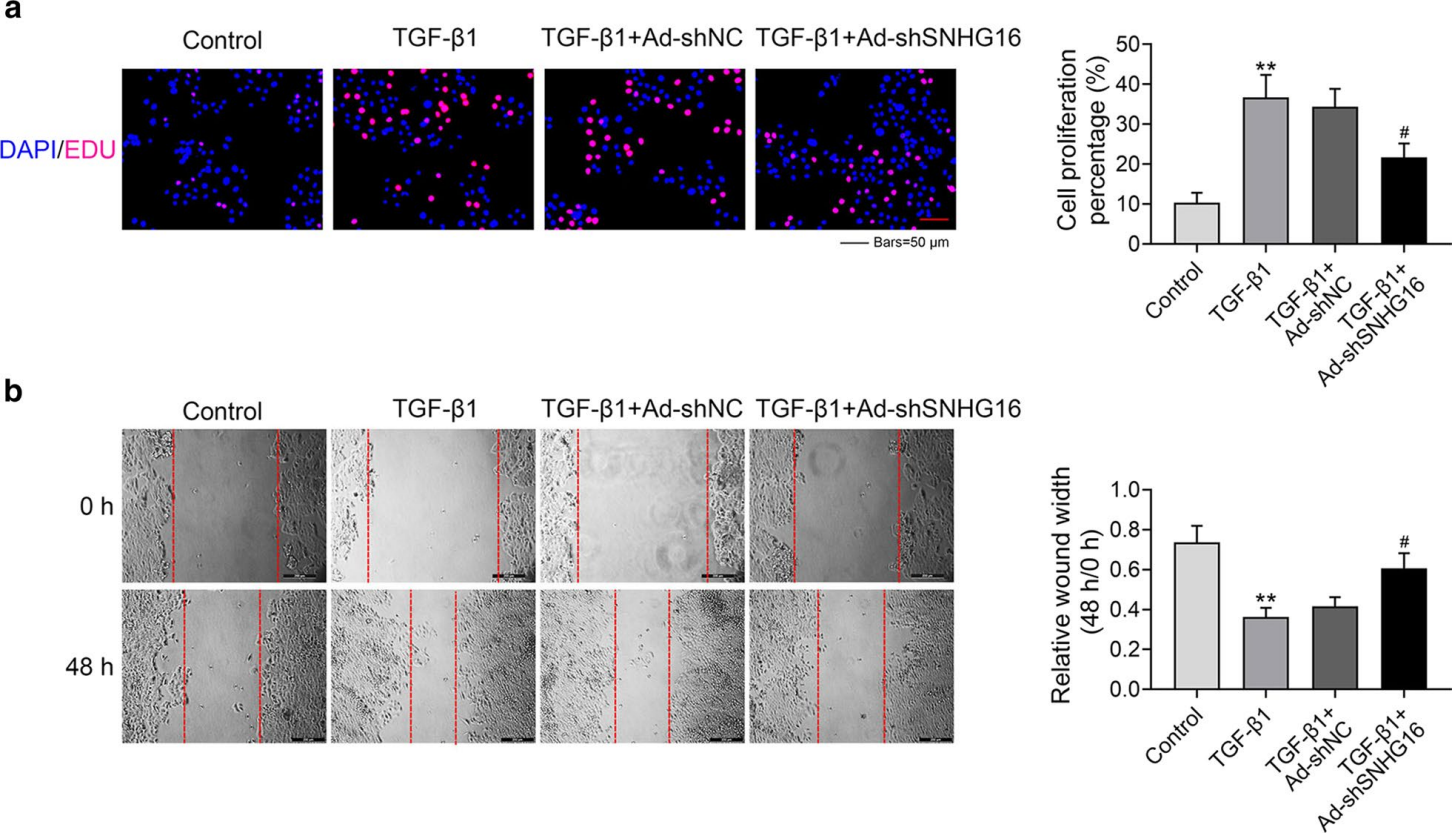
Knockdown of SNHG16 suppressed the proliferation and migration of lung fibroblast cells. The role of knockdown of SNHG16 expression on the proliferation (**a**) and migration (**b**) abilities of TGF-β1 treated lung fibroblast cells were checked by EdU staining and wound healing assay, respectively. Data were expressed as mean ± SD. **P < 0.01 was control, ^##^*P* < 0.01 vs TGF-β1 + Ad sh-SNHG16, represent statistically difference

Further, the effect of silencing SNHG16 on lung fibroblast cells was detected by Edu staining and wound healing assay. TGF-β1 treated lung fibroblast cells were transfected with sh-SNHG16 or its control sh-NC. As shown in Fig. 4a, the percentage of cell proliferation was dramatically increased upon the treatment of TGF-β1 (*p* < 0.01), while this effect was reversed by knockdown of SNHG16 (*p* < 0.05). In addition, wound healing assay was conducted to identify the migration ability of lung fibroblast cells after transfection of sh-SNHG16 (Fig. 4b). Results demonstrated that treatment with TGF-β1 could promote the migration of lung fibroblast cells (*p* < 0.01). However, after transfected with sh-SNHG16, the migration rate was significantly decreased (*p* < 0.05). These data suggested that knockdown of SNHG16 could suppress the proliferation and migration of lung fibroblast cells.

### SNHG16 regulated the expression of miR-455-3p

**Fig. 5:**
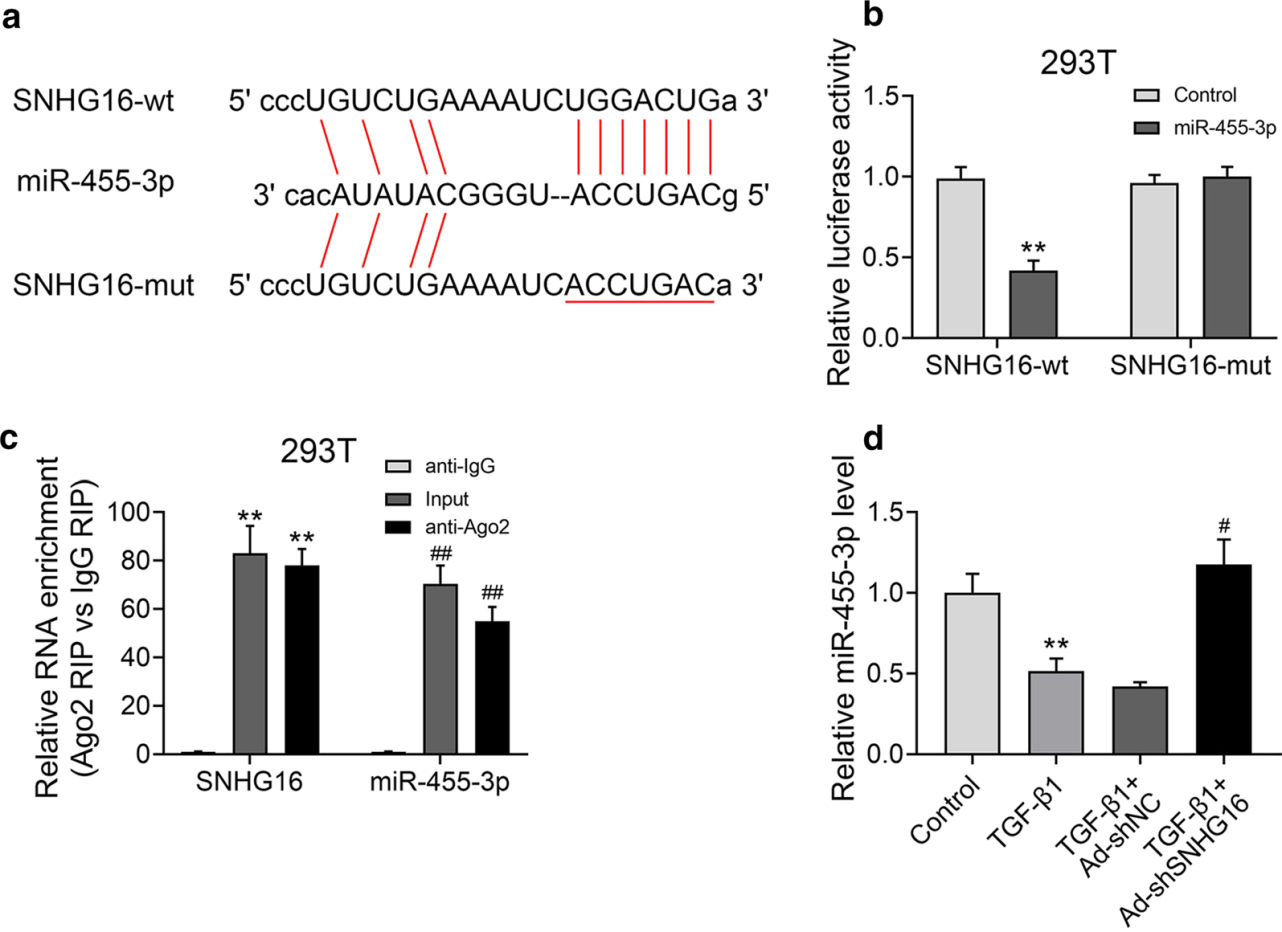
SNHG16 regulated the expression of miR-455-3p. **a** SNHG16 mRNA wide-type (SNHG16 WT) and the mutated-type (SNHG16 MUT) in the miR-455-3p binding sites were shown. **b** Luciferase activity of HEK293T cells co-transfected with miR-455-3p mimics or NC mimics and luciferase reporters containing SNHG16 WT or SNHG16 MUT transcript were determined by dual-luciferase reporter assays. **c** Endogenous miR-455-3p precipitated by AGO2 upon overexpression of SNHG16 was determined by RIP assay. **d** Relative miR-455-3p was in different groups determined by qRT-PCR. Data were expressed as mean ± SD. **P < 0.01 vs control or anti-IgG; ^#^*P* < 0.05 vs TGF-β1 + Ad sh-SNHG16; ^##^*P* < 0.01 vs anti-IgG, represent statistically difference

Next, miR-455-3p was predicted as the potential bio-target of SNHG16 by Starbase (http://starbase.sysu.edu.cn) and the targeting relationship was further confirmed by dual-luciferase reporter assay. The biding site of 3′ UTR region of SNHG16 and miR-455-3p was shown in Fig. 5a. Then, plasmids containing the potential miR-455-3p binding sites, SNHG16 wild-type (WT) or mutated (MUT) were co-transfected with miR-NC or miR-455-3p mimic. We found that relative luciferase activity was significantly lower in SNHG16 WT group than that in SNHG16 MUT group (Fig. 5b, p < 0.01), suggesting that miR-455-3p could be a direct target of SNHG16. Further, the results of RIP experiments showed that compared with control IgG, SNHG16 was preferentially enriched in miRNA ribonucleoprotein complexes containing Ago2 (Fig. 5c, p < 0.01). In addition, the expression of miR-455-3p was determined by qRT-PCR in different groups. In TGF-β1 treated group, the expression of miR-455-3p was remarkably decreased (Fig. 5d, p < 0.01), while knockdown of SNHG16 reversed the tendency (Fig. 5d, p < 0.05). Together, these data confirmed that miR-455-3p was a direct target of SNHG16.

### SNHG16 regulated expression of Notch2 by targeting miR-455-3p

**Fig. 6:**
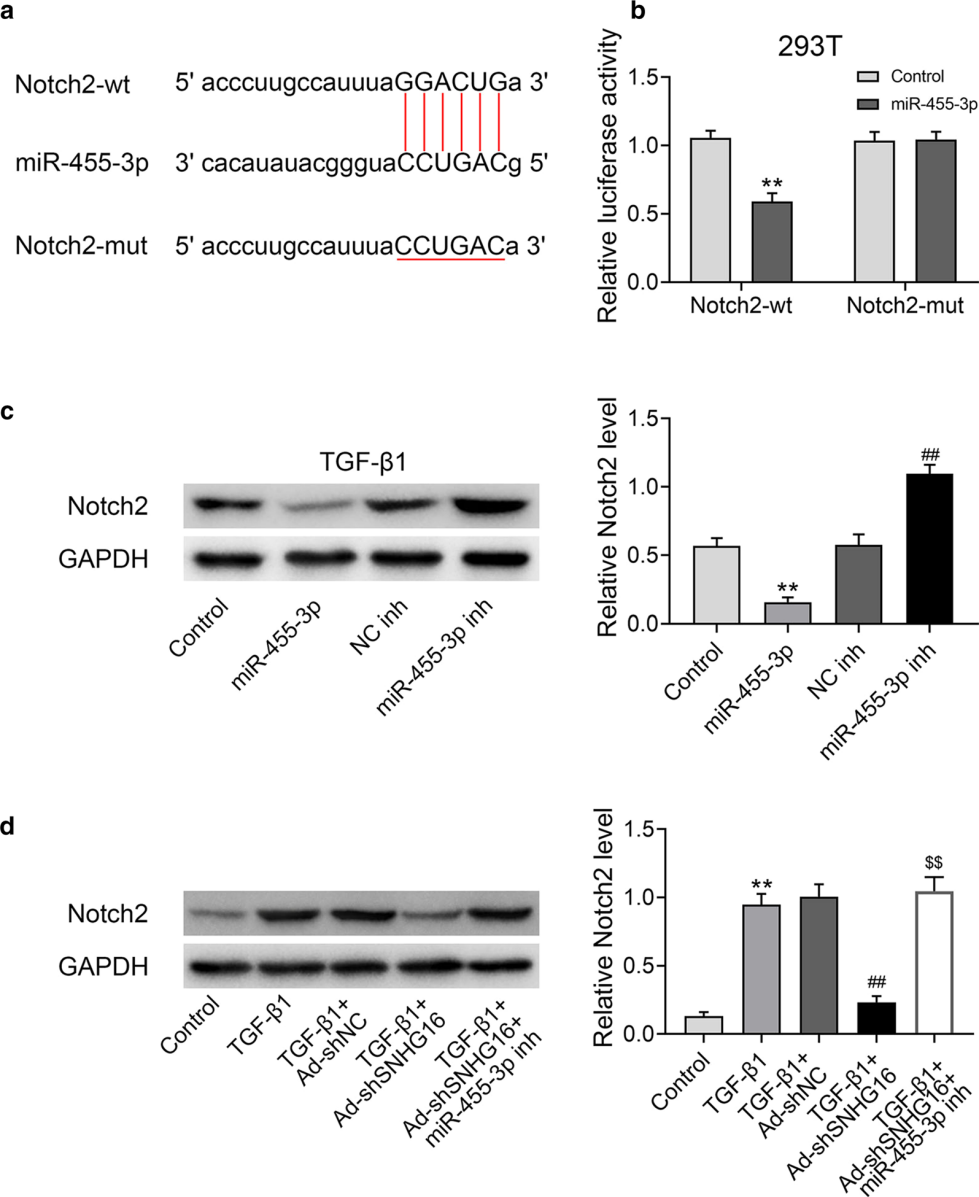
SNHG16 regulated expression of Notch2 by targeting miR-455-3p. **a** Notch2 mRNA wide-type (Notch2 WT) and the mutated-type (Notch2 MUT) in the miR-455-3p binding sites were shown. **b** Luciferase activity of HEK293 cells co-transfected with miR-455-3p mimics or NC mimics and luciferase reporters containing Notch2 3′-UTR WT or Notch2 3′-UTR MUT transcript were determined by dual-luciferase reporter assays. **c** The expression level of protein Notch2 was detected in miR-455-3p mimic, inhibitor and their control groups (control, NC-inh) by western blot. **d** The expression level of protein Notch2 were analyzed by western blot in TGF-β1 treated lung fibroblast cells which co-transfected with sh-SNHG16 or its control (sh-NC) and miR-455-3p inhibitor or its control (NC-inh). Data were expressed as mean ± SD. **P < 0.01 vs control; ^##^*P* < 0.01 vs NC-inh or TGF-β1 + Ad sh-NC; ^$$^*P* < 0.01 vs TGF-β1 + Ad sh-SNHG16, represent statistically difference

The mechanism of miR-455-3p was further explored. By using Starbase (http://starbase.sysu.edu.cn), Notch2 was shown as a potential target of miR-455-3p (Fig. 6a). Then, Notch2 wild-type (WT) plasmid or mutated (MUT) plasmid containing binding sites of these two factors, was co-transfected with miR-NC or miR-455-3p mimic. Results showed that the relative luciferase activity was dramatically decreased in Notch2 WT group (Fig. 6b, p < 0.01), indicating that Notch2 was a target of miR-455-3p. Then, the protein expression of Notch2 in different groups was detected by western blotting (Fig. 6c, d). Firstly, lung fibroblast cells were transfected with miR-455-3p mimics, inhibitors and their controls. After treatment with TGF-β1, the expression of Notch2 was identified. As shown in Fig. 6c, the expression of Notch2 was significantly decreased in miR-455-3p overexpression group, while increased in miR-455-3p inhibition group (*p* < 0.01). Moreover, the expression of Notch2 was up-regulated under treatment of TGF-β1 (Fig. 6d, p < 0.01), while after transfected with sh-SNHG16, the expression of Notch2 was significantly down-regulated (*p* < 0.01). However, the effect of sh-SNHG16 can be reversed by inhibiting miR-455-3p (*p* < 0.01). Moreover, functional experiments of how SNHG16 affects cell proliferation and migration via miR-455-3p was conducted on TGF-β1 treated lung fibroblast cells which co-transfected with sh-SNHG16 or sh-NC and miR-455-3p or miR-NC. As shown in Additional file 1: Fig. S1, knockdown of SNHG16 could inhibit the proliferation and migration, and this effect was reversed by inhibitory of miR-455-3p. Taken together, these results demonstrated that SNHG16 regulated expression of Notch2 by targeting miR-455-3p.

### SNHG16 promoted fibrogenesis by regulating expression of Notch2

**Fig. 7:**
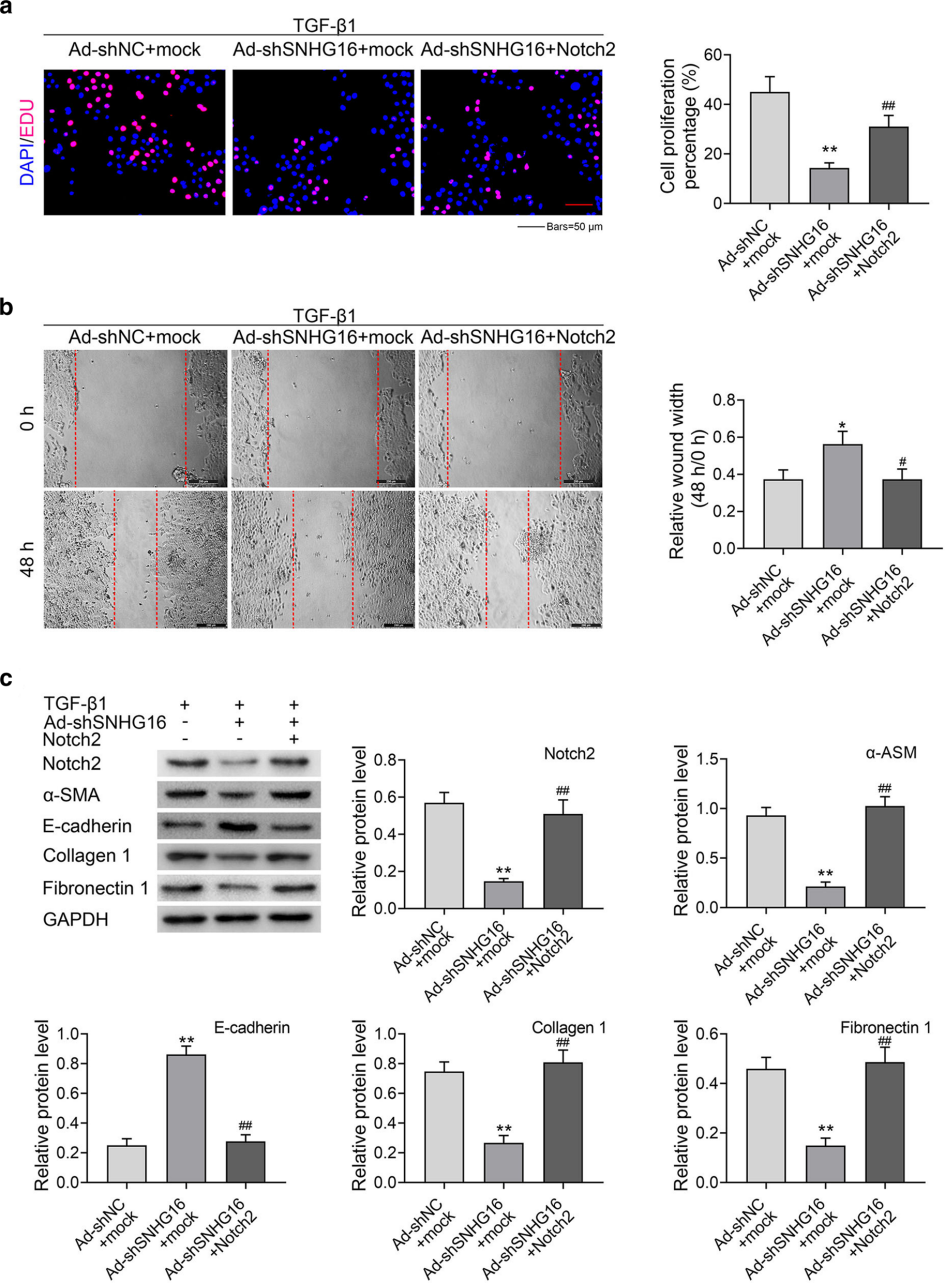
SNHG16 promoted fibrogenesis by regulating expression of Notch2. **a** TGF-β1 treated lung fibroblast cells which co-transfected with sh-SNHG16 or sh-NC and Notch2 or mock were used for experiments: The proliferation (**a**) and migration (**b**) abilities of TGF-β1 treated lung fibroblast cells in different groups were checked by EdU staining and wound healing assay, respectively. **c** The protein expression of α-SMA, E-cadherin, collagen 1 and fibronectin 1 were determined by western blot. Data were expressed as mean ± SD. **P < 0.01 vs Ad-shNC + mock; ^##^*P* < 0.01 vs Ad-sh-SNHG16 + mock, represent statistically difference

Finally, the effects of Notch2 and SNHG16 on proliferation and migration of lung fibroblast cells were clarified. To this end, overexpression plasmid pcDNA3.1-Notch2 (Notch2) or its negative control plasmid (mock) was co-transfected with SNHG16 knockdown plasmid (sh-SNHG16) or its negative control plasmid (sh-NC). Results revealed that knockdown of SNHG16 significantly inhibited the proliferation (Fig. 7a) and migration (Fig. 7b) of lung fibroblast cells, while this effect was compromised by overexpression of Notch2 (*p* < 0.01, *p* < 0.05). In addition, the expression of Notch2, α-ASM, E-cadherin, Collagen 1 and Fibronectin 1 was detected in different groups by western blotting. As shown in Fig. 7c, the expression of Notch2, α-ASM, Collagen 1 and Fibronectin 1 was remarkably suppressed by knockdown of SNHG16 (*P* < 0.01), while overexpression of Notch2 reversed this effect (*p* < 0.01). On the contrary, the expression of E-cadherin showed different tendency (*p* < 0.01). These data indicated that SNHG16 promoted fibrogenesis by regulating expression of Notch2.

## Discussion

With the rapid development of deep transcriptome sequencing technology, research on non-coding RNAs has grown increased exponentially [23]. Although in some cases, without protein coding ability, ncRNAs seem lack of bio-function, more and more evidence confirmed that they play critical roles in controlling gene expression through a variety of mechanisms, e.g. targeting transcripts [24], affecting splicing function [25], targeting cis-acting promoter RNAs [26]. In our study, we found that lncRNA SNHG16 was highly expressed in BLM-induced pulmonary fibrotic animal tissues. Moreover, lung fibrogenesis can be affected by expression of SNHG16. We also explored the potential mechanism of how SNHG16 exerted its role, and we found that SNHG16 directly targeted miR-455-3p, thereby decreasing protein levels of Notch2. Our data indicated that lncRNA SNHG16 could promote pulmonary fibrosis by targeting miR-455-3p to regulate the Notch2 pathway.

Regarding the pathogenesis of IPF, repeated micro-injury of the alveolar epithelium over time is considered to be the first trigger of the maladaptive repair process [27]. Subsequently, aberrant epithelial-mesenchymal crosstalk leads to an imbalance between profibrotic and antifibrotic mediators, followed by fibroblasts, myofibroblasts proliferation and accumulation of extracellular matrix [28, 29]. In these process, epithelial mesenchymal transition (EMT) has been considered to be the milestones of fibrogenesis [30]. Previous studies confirmed that lncRNA SNHG16 acts as a promotor of EMT process in variety of cancer types, e.g. glioma [31], esophagus cancer [21], cervical cancer [32], and gastric cancer [20]. Therefore, our hypothesis was that lncRNA SNHG16 may also play important roles in lung fibrogenesis. To this end, we not only used BLM-induced pulmonary fibrotic animal model and TGF-β1-induced pulmonary fibrotic cell model in vitro, but also employed different series of transfected lung fibroblast cells systems to explore the underlying molecular mechanisms. Results demonstrated that knockdown of SNHG16 could attenuate BLM-induced pulmonary fibrosis, and suppress the proliferation and migration process of lung fibroblast cells. These findings suggested that SNHG16 palys an accelerator of lung fibrogenesis which is in line with previous studies.

Increasing studies have reported that, in some cases, lncRNA and miRNA can cross-talk in a competing endogenous RNA (ceRNA) process [33]. In this condition, lncRNAs might act as “sponge”, thus, miRNAs are isolated from their targeted mRNAs, leading to the expression changes of their target genes [34]. In our study, by using dual-luciferase reporter assay and RIP assay, we found that SNHG16 was able to bind to miR-455-3p. Moreover, the expression of miR-455-3p can be regulated by SNHG16. Furthermore, Notch2 was confirmed to be a target of miR-455-3p, and can be regulated by both SNHG16 and miR-455-3p to affect the process of lung fibrogenesis.

As important candidates in our study, miR-455-3p and Notch2 have also been confirmed to be involved in pathological progressions of lung fibrogenesis in many published studies. Previous study showed that miR-455-3p could act as a fibrogenesis suppressor. It may exert its role by potentially binding to Bax gene to suppress apoptosis of alveolar epithelial cells [35]. Notch2 is one of the most important members of Notch receptor family, composed of 34 exons and encoded by 2471 amino acids [36]. Studies have demonstrated that Notch2 can be detected in bronchiolar epithelial cells [37], and plays important roles in small-cell lung cancer [38]. In IPF study field, Notch2 was reported to be involved in immune system [39]. More evidence regarding mechanisms of Notch2 in IPF are greatly needed to valid the current results.

## Conclusions

Taken together, the results from this study demonstrated that lncRNA SNHG16 served as an essential regulator in lung fibrosis. Moreover, SNHG16 functioned as a miR-455-3p sponge, thereby positively regulating Notch2 expression by binding miR-455-3p. Our findings might provide a novel insight into pathological of fibrosis. However, there are remaining puzzles need to be solved, such as whether the expression of SNHG16 is related with prognosis of patients with IPF, and how SNHG16 exerts its role in different stage of IPF. Therefore, in future, human-related clinical studies will be required.

## Supplementary Information

**Additional file 1: Fig. S1.** SNHG16 affects cell proliferation and migration via miR-455-3p. TGF-β1 treated lung fibroblast cells which co-transfected with sh-SNHG16 or sh-NC and miR-455-3p or miR-NC were used for experiments: (A) The proliferation of TGF-β1 treated lung fibroblast cells in different groups were checked by EdU staining. (B) The migration abilities of TGF-β1 treated lung fibroblast cells in different groups were investigated by wound healing assay.

## Data Availability

All data generated or analyzed during this study are included in this published article.

## Abbreviations

IPF: Idiopathic pulmonary fibrosis
lncRNAs: Long non-coding RNAs
BML: Bleomycin
qRT‐PCR: Quantitative real-time polymerase chain reaction
RIP: RNA immunoprecipitation assay
TGF-β: Transforming growth factor-β
ECM: Extracellular matrix
ncRNAs: Non-protein coding RNAs
EMT: Epithelial-mesenchymal transition
