# Supplementary figures and images for: LncRNA SNHG16 promotes pulmonary fibrosis by targeting miR-455-3p to regulate the Notch2 pathway
Source: Respir Res. 2021 Feb 6;22:44. doi: 10.1186/s12931-021-01632-z (PMC7866661; doi:10.1186/s12931-021-01632-z)

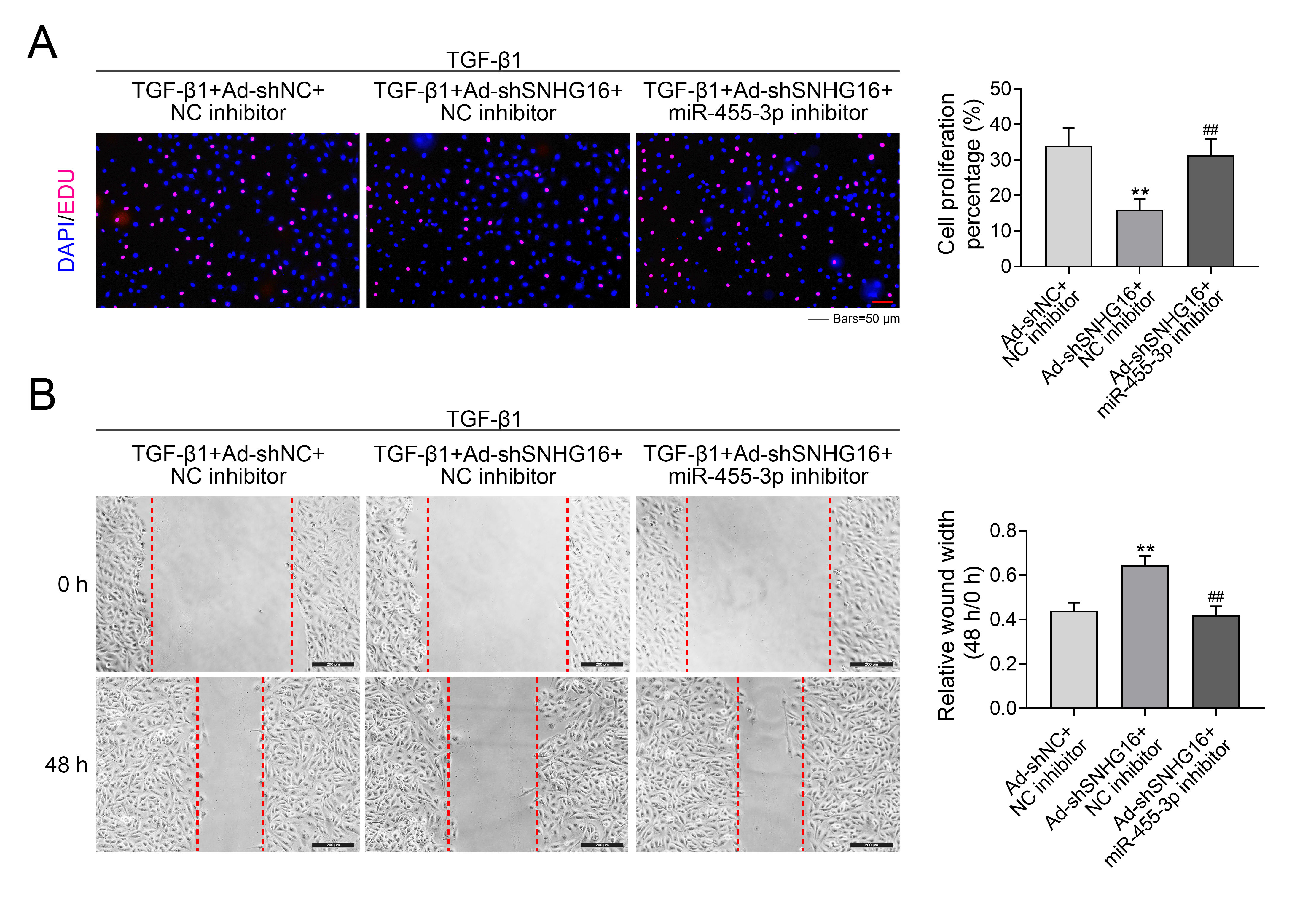

Supplement: Supplementary file 1 — Additional file 1: Fig. S1. SNHG16 affects cell proliferation and migration via miR-455-3p. TGF-β1 treated lung fibroblast cells which co-transfected with sh-SNHG16 or sh-NC and miR-455-3p or miR-NC were used for experiments: (A) The proliferation of TGF-β1 treated lung fibroblast cells in different groups were checked by EdU staining. (B) The migration abilities of TGF-β1 treated lung fibroblast cells in different groups were investigated by wound healing assay. [file 12931_2021_1632_MOESM1_ESM.jpg]
